# Facile Preparation of Tunicate-Inspired Chitosan Hydrogel Adhesive with Self-Healing and Antibacterial Properties

**DOI:** 10.3390/polym13244322

**Published:** 2021-12-10

**Authors:** Xiang He, Ruyue Liu, Huiqing Liu, Ruixiao Wang, Zhenhao Xi, Yixiang Lin, Jie Wang

**Affiliations:** 1 State Key Laboratory of Chemical Engineering, East China University of Science and Technology, Shanghai 200237, China; y30190862@mail.ecust.edu.cn (X.H.); Y30190901@mail.ecust.edu.cn (R.L.); chen7410@foxmail.com (H.L.); Y30200220@mail.ecust.edu.cn (R.W.); 19000157@mail.ecust.edu.cn (Y.L.); 2 Shanghai Key Laboratory of Multiphase Materials, Chemical Engineering, East China University of Science and Technology, Shanghai 200237, China

**Keywords:** facile preparation, tunicate-inspired, chitosan, hydrogel, tissue adhesive

## Abstract

In order to replace traditional wound treatments such as sutures, tissue adhesives with strong wet tissue adhesion and biocompatibility have attracted more attention to the applications of non-invasive wound closure. Herein, inspired by tunicate adhesive protein, a series of 2,3,4-trihydroxybenzaldehyde (TBA)-modified chitosan hydrogels (CS-TBA-Fe) were prepared by easily mixing the solutions of chitosan-FeCl_3_ and TBA via the Schiff-base reaction and the coordination between Fe^3+^ and pyrogallol groups. The gelation time was greatly shortened to only several seconds after induced even trace Fe^3+^. The hydrogel (CS-TBA-Fe) exhibited ~12-fold enhanced wet tissue adhesion strength (60.3 kPa) over the commercial fibrin glue. Meanwhile, the hydrogel also showed robust adhesion to various substrates such as wood, PMMA, and aluminum. The swelling ratio and rheological property can be simply controlled by changing the concentrations of chitosan, TBA, and Fe^3+^. Moreover, the hydrogel displayed a rapid and highly efficient self-healing ability and an excellent antibacterial activity against *E. coli*. The overall results show that the CS-TBA-Fe hydrogel with enhanced wet adhesiveness will be a promising tissue adhesive material.

## 1. Introduction

Skin is the first barrier that protects the body from external injuries and bacterial infections, but it is susceptible to external injuries and form irregular wounds [1]. Because of the increasing number of surgical procedures every year, surgery wound healing is under great pressure with a high risk of wound infection and the secondary wound injury [2]. Tissue adhesive is expected to replace sutures and bone needles as a wound dressing biomaterial because of it is easy to handle, less invasive, and high adaptable to irregularly shaped wounds [3,4]. For such applications, synthetic (i.e., poly (ethylene glycol) bioadhesives [5] and cyanoacrylated glues [6]), proteinic (i.e., heterologous fibrin glues [7]), polysaccharides adhesives (i.e., chitosan adhesive [8] and hyaluronic acid adhesive [9]), and hybrid tissue adhesives (i.e., gelatin−resorcinol−formaldehyde [10]) are currently available commercially. However, weak adhesive strength on wet biological tissue, slow gelation time, lack of antibacterial properties and self-healing ability are still major challenges for tissue adhesives [11,12]. Therefore, the study of new biocompatible tissue adhesives with tight adhesion and antibacterial property is of great significance and urgency [13,14].

Chitosan (CS), as a naturally derived polysaccharide, has good biocompatibility, biodegradability, and intrinsic mucoadhesive properties [15,16]. Moreover, previous studies convinced that the hemostatic effects and antibacterial activity against *Escherichia coli* and *Staphylococcus aureus* of chitosan [17,18]. For these reasons, chitosan has attracted great attention in the field of tissue adhesives [19,20].

Inspired by the mussels’ outstanding adhesive activity to various substrates in moisture environment, catechol, derived from 3,4-dihydroxyphenylalanine (DOPA), has been incorporated into hydrogels, which usually have good adhesive ability to wet soft tissues [21]. Similarly, tunicate-inspired wet adhesive hydrogels are prepared based on pyrogallol derived from 3,4,5-trihydroxyphenylalanine (TOPA) [22,23]. Catechol or pyrogallol modified polymer hydrogels were designed by oxidizing polyphenol groups to form covalent crosslinking, or ion coordination to form ionic crosslinking [24]. Based on polyphenol chemistry, these hydrogels can connect with tissues or other materials by coordination bonds, covalent bonds, hydrogen bonds, π–π, cation–π, and other forces formed [25]. Recently, many mussel-inspired hydrogels have been reported; however, tunicate-inspired hydrogel with multifunctional properties are still rare. Pyrogallol contains more hydroxyl groups than catechol, which is expected to form hydrogels with excellent underwater adhesion and the self-healing ability [26,27]. Therefore, it is necessary to construct a tunicate-inspired chitosan-based hydrogel with multifunctional properties include robust wet adhesive ability, antibacterial activity and self-healing property.

In this paper, inspired by tunicate, a novel chitosan-based one-step in situ-forming hydrogel (CS-TBA-Fe) was designed by simply mixing chitosan-Fe^3+^ solution (CS-Fe) with 2,3,4-trihydroxybenzaldehyde (TBA), an analog of TOPA. Based on the Schiff-base reaction between chitosan and TBA and the dual crosslinking by irreversible oxidation of pyrogallol and reversible coordination between Fe^3+^ and pyrogallol groups, a hydrogel with wet adhesive ability can be instantly prepared. The effect of the concentration of chitosan, TBA and Fe^3+^ on the gelation time, swelling ratio, mechanical property, and adhesive strength were systematically investigated. The introduction of pyrogallol moieties is supposed to give the hydrogel good antibacterial and self-healing abilities. Thus, the prepared CS-TBA-Fe hydrogel have many potential applications for wound closure, healing and tissue engineering.

## 2. Materials and Methods

### 2.1. Materials

Chitosan (CS, BR, 90% deacetylated) was purchased from Nantongfeiyu Biotech Co. (Nantong, China) 2,3,4-Dihydroxybenzaldehyde was supplied by Shanghai Aladdin Bio-Chem Technology Co., Ltd. (Shanghai, China) Acetic acid was obtained from Shanghai Macklin Biochemical Co., Ltd. (Shanghai, China) Iron(III) chloride hexahydrate was purchased from Sinopharm Chemical Reagent Co., Ltd.( Shanghai, China) All other reagents were of analytical grade and were used without further purification.

### 2.2. Preparation of the Hydrogels

CS-TBA-Fe hydrogels were prepared by the one-step in situ method through the Schiff-base reaction and ion coordination interactions. First, an appropriate mass of chitosan was dissolved in a certain concentration of FeCl_3_ solution (CS-Fe) at room temperature, and then a desired amount of TBA in deionized water was added to the CS-Fe solution, homogenized by mechanically stirring. The brown hydrogel was formed immediately, which was named the CS-TBA-Fe-x-y-z hydrogel, where x, y, and z represent the chitosan concentration in mass percentage (wt%), and the molar concentration of TBA (M) and Fe^3+^ ions (mM), respectively.

The Fourier transform infrared (FT-IR) spectra of chitosan and CS-TBA were recorded on an FT-IR spectrometer (Nicolet 6700, Thermo Nicolet Corporation., Madison, WI, USA).

### 2.3. Gelation Time

Gelation time of CS-TBA-Fe hydrogel was measured by the vial tiling method. Hydrogel samples with different concentration of chitosan polymer, TBA and Fe^3+^ were prepared in room temperature, then monitored by inversing test tubes every 5 s. The gelation time was defined as the time at which no flow was observed in the vial.

### 2.4. Swelling Ratio

The swelling behavior of the CS-TBA-Fe hydrogels was assayed by gravimetric analysis. The CS-TBA-Fe hydrogels were freeze-dried and weighed as the dry gel weight (W0) and then immersed in 1% acetic acid solution. At specific intervals, the swollen hydrogels were weighed after gently removing the adhering solutions using filter paper and recorded as Wt. The swelling ratio was calculated using (Wt − W0)/W0. The equilibrium swelling ratio was determined until hydrogels had no further weight change. All experiments were performed in triplicate.

### 2.5. Rheological Properties

Viscoelastic properties of CS-TBA-Fe hydrogels were characterized by an Anton-Paar MCR 501 rheometer(Anton Paar, Graz, Austria) using a parallel plate geometry (5 mm plate diameter, 0.5 mm sample gap) in oscillation mode at 37 °C. To determine the linear viscoelastic region (LVR), the oscillation strain sweep was performed at a strain range from 0.1 to 100% at a constant 1 Hz frequency. Further, frequency sweep was in the range of 0.1 Hz to 50 Hz, and temperature sweep was from 25 °C to 55 °C to assess the stability of hydrogels. Defaulting to the basic test condition as strain = 1%, frequency=1 Hz if no special instructions are given.

### 2.6. Self-Healing Test

The self-healing ability of hydrogels was evaluated by the recycle stresses test at 1 Hz by an Anton-Paar MCR 501 rheometer(Anton Paar, Graz, Austria). Specifically, after a weak 1% stress of amplitude oscillatory stress for 250 s, the stress would increase to 500% suddenly and remain for 100 s.

### 2.7. Tissue Adhesive Strength

The tissue adhesion strength of CS-TBA-Fe hydrogels were investigated by lap shear tests [28], the wet porcine skin was widely used as the substrate materials to simulate the real nature of human tissue. Porcine skin substrates (2 mm thick) were cut into pieces with 25 × 25 mm and dehydrated in PBS buffer solution (pH = 7.4) for 2 h. Then, porcine skins were attached to the aluminum plate glued by commercial cyanoacrylate glue. The pre-gel solution of CS-Fe (300 µL) was applied on one porcine skin piece, and TBA solution (200 µL) was coated onto the surface of another porcine piece uniformly. Then the two plates were stacked up quickly and pressed tightly with clips at 37 °C for 2 h in the PBS solution. It was evaluated by a universal testing machine (UTM, HengYi, HY-0580, Shanghai Hengyi Precision Instrument Co., Ltd, Shanghai, China) at room temperature with a 3000 N loading cell at a strain speed of 5 mm/min in accordance with the ASTM F2255-05 standards. Each measurement was repeated at least three times. Adhesion strength was calculated on a formula of dividing the maximum load force by overlapping contact area.

The adhesion strength of CS-TBA-Fe hydrogel to other different substrate materials like plastic and glass was also tested. The substrate materials were glued with cyanoacrylate glue on aluminum bars, the test method of adhesion strength was same as porcine skin specimens.

### 2.8. In Vitro Antibacterial Test

The antibacterial activity of CS-TBA-Fe hydrogels was evaluated on *E. coli* (Gram-negative bacteria) [29]. First, ~150 mg of chitosan, TBA, Fe solution were put into culture dish, respectively, and sterilized by UV light. The difference of optical density (OD) between the CS-TBA-Fe hydrogels and the control samples could determine the antibacterial ability against *E. coli* roughly by Abssample/Abscontrol × 100%. Fifty microliters of *E. coli* bacterial suspension was incubated with the hydrogel in a thermostatic shaker at 37 °C, at 200 rpm for 6 h. The resulting bacterial suspension was diluted with sterilized PBS buffer solution, spread on the lysogeny broth medium and cultured for 18 h to measure the number of colony-forming units and the concentration of bacterial solution.

## 3. Results

### 3.1. Synthesis and Characterization of CS-TBA-Fe Hydrogel

**Figure 1:**
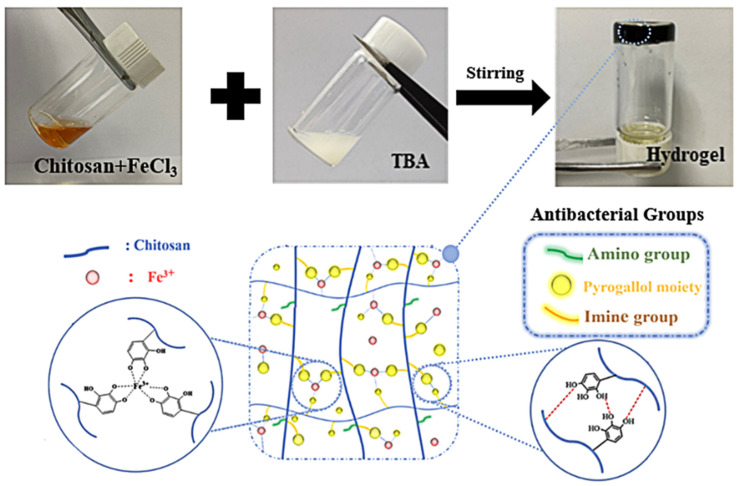
Schematic illustration of the formation of CS-TBA-Fe hydrogel by mixing CS-Fe and TBA solutions.

The antibacterial CS-TBA-Fe hydrogel was prepared by a simple and mild method of mixing the CS-Fe solution and TBA solution by magnetic stirring (Figure 1). During the mixing process, the Schiff-base reaction was carried out between the aldehyde groups of TBA and the amino groups of chitosan.

**Figure 2:**
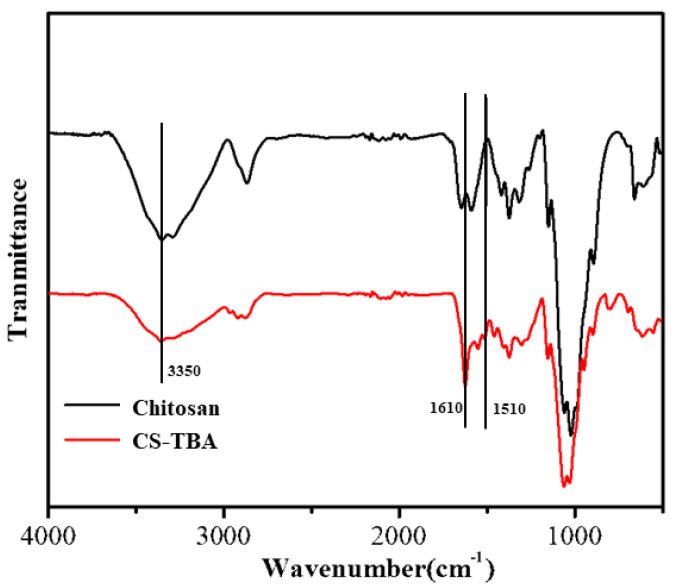
Infrared spectra of chitosan and CS-TBA polymer.

In order to confirm the Schiff-base reaction between TBA and the amino groups of chitosan, the TBA-grafted chitosan (CS-TBA) was prepared and characterized by FTIR spectra as shown in Figure 2. The chitosan displayed a O-H and a N-H stretching peak at 3350 cm^−1^, a C-H stretching peak at 2870 cm^−1^, an amide I region of chitosan stretching peak (the C=O stretching peak of acetyl group) at 1645 cm^−1^, an amide II region vibration peak at 1590 cm^−1^ (a N-H bending peak and a C-N stretching peak), and a typical stretching vibration peak of C-O at 1070 cm^−1^ and 1020 cm^−1^. The CS-TBA spectrum displayed a stronger stretching peak at 1610 cm^−1^, which is due to the superposition of the C=N and the amide region vibrational peak in the product. A stretching peak at 1510 cm^−1^ was also discovered, which was attributed to the hydroxyl group on the pyrogallol. The weaken of the peak at 3350 cm^−1^ of N-H for CS-TBA was also observed, which confirmed the consuming of the amino groups in chitosan during the Schiff-base reaction. The presence of amino group, pyrogallol moiety and imine group is supposed to give excellent antibacterial capability to the CS-TBA-Fe hydrogel.

### 3.2. The Gelation Time of CS-TBA-Fe Hydrogel

**Figure 3:**
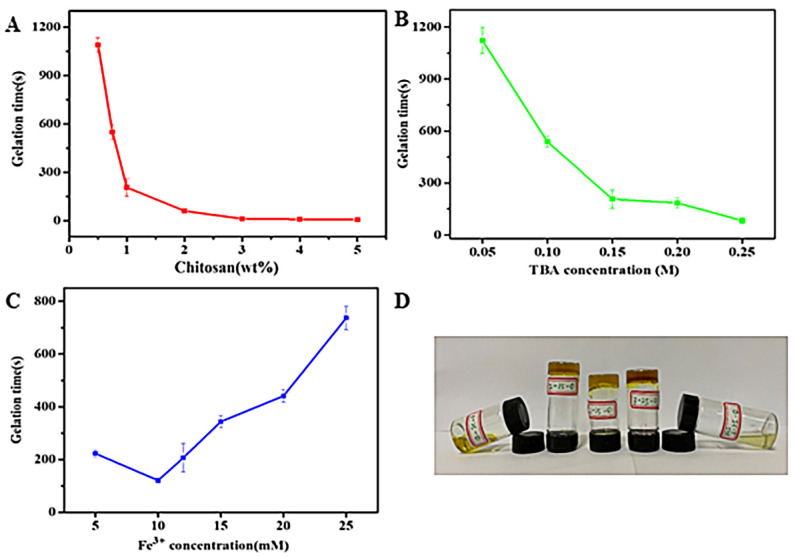
Gelation time of CS-TBA-Fe hydrogels varies with concentration of chitosan (**A**), TBA (**B**), and Fe^3+^ (**C**), and the photos of the mixtures of chitosan and TBA without Fe^3+^ (**D**).

The effect of the concentration of chitosan, TBA, and Fe^3+^ on the gelation time of CS-TBA-Fe hydrogels is shown in Figure 3. When the TBA and Fe^3+^ concentrations were kept at 0.15 M and 12 mM, the gelation time decreased from 1090 s to 10 s with the increase of chitosan concentration from 0.5 wt% to 4 wt%. Similarly, when the chitosan and Fe^3+^ concentrations were kept at 1 wt% and 12 mM, the gelation time decreased from 1112 s to 83 s as increasing the concentration of TBA from 0.05 M to 0.25 M. This was in consistent with that the high cross-linking degree results in short gelation time. However, when the chitosan and TBA concentrations were kept at 1 wt% and 0.15 M, the gelation time decreased at low Fe^3+^ concentration while increased at a higher Fe^3+^ concentration (>10 mM). At low concentration, Fe^3+^ would crosslink with the hydroxyl groups of pyrogallol moieties for multiple coordination, which causing faster gelling than single interaction in higher Fe^3+^ concentration. As shown in Figure 3C, CS-TBA-Fe hydrogels could form gel with only 0.05 mM Fe^3+^, so that the contribution of other crosslinking interactions to the gelation of CS-TBA without Fe^3+^ was further investigated. As shown in Table 1, CS-TBA-Fe-2-15-0, CS-TBA-Fe-2-25-0 and CS-TBA-Fe-3-25-0 could crosslink without Fe^3+^ while the CS-TBA-Fe-1-25-0 could not gel because of the low concentration of chitosan. When CS-TBA-Fe-3-25-0 prepared in 0.2 M HCl solution, the Schiff reaction between TBA and chitosan was prevented, and it failed to form gel, indicating that the hydrogen bonding between pyrogallol moieties grafted in chitosan plays a major role in the gelation process.

### 3.3. Swelling Capacities of CS-TBA-Fe Hydrogels

**Figure 4:**
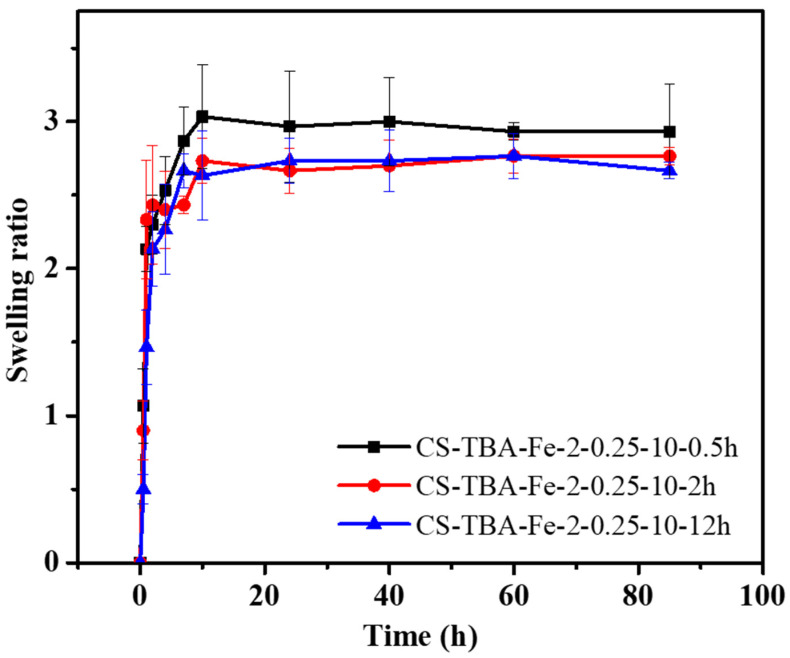
Swelling test of CS-TBA-Fe hydrogels with different curing time.

The swelling capacity is a general feature of the hydrogel that reflects polymer construction, the degree of crosslinking within the hydrogel and the interaction between hydrogel polymer and aqueous solutions. In this paper, the swelling degree can reflect the stability of CS-TBA-Fe hydrogels formed by dual crosslinking interactions of pyrogallol oxidation and Fe^3+^ coordination. The swelling capacities of the CS-TBA-Fe hydrogels were investigated by calculating the weight change from the initial dry gels to the wet gels under 1% acetic acid solution at 37 °C during the specific time intervals. As shown in Figure 4, the swelling rate of the CS-TBA-Fe hydrogels increased rapidly in the first 2 h. Then, the hydrogels reached the swelling equilibrium in about 7 h, and they had no obvious disintegration even for 85 h. Therefore, CS-TBA-Fe hydrogels can maintain good cross-linking stability in aqueous environment for a long time.

### 3.4. Rheological Behavior of CS-TBA-Fe Hydrogels

**Figure 5:**
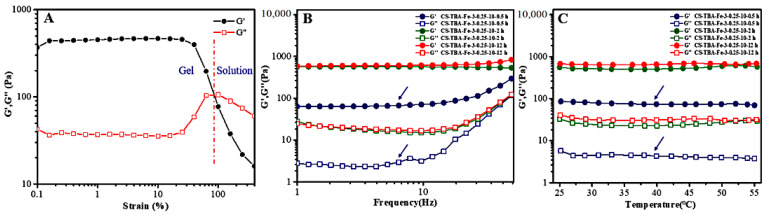
Linear viscoelastic region of CS-TBA-Fe hydrogels (**A**), and the frequency sweep (**B**) and temperature sweep **(C**) tests with different curing time.

The linear viscoelastic region, frequency stability, and temperature stability of CS-TBA-Fe hydrogels were determined by oscillation mode of the rheometer as shown in Figure 5. The concentration of chitosan, TBA, and Fe^3+^ was 3 wt%, 0.25 M, and 10 mM, and the curing time was 0.5 h, 2 h, and 12 h, respectively. When the test strain was between 0.1% and 20%, the change of the G’ and G’’ was small (Figure 5A), which indicating that this strain range within the linear viscoelastic region of the sample. In the frequency scan test and temperature scan test, the strain will be set to 1% to ensure that the test conditions stay within the linear viscoelastic region of CS-TBA-Fe hydrogel. The stabilities of CS-TBA-Fe hydrogels was investigated by frequency sweep tests in 37 °C as changing the curing time. As shown in Figure 5B, the G’ of CS-TBA-Fe-3-0.25-10 hydrogels in different curing time was always higher than G’’ in the tested frequency range 1 Hz to 50 Hz. In addition, G’ of the hydrogels was gradually increase as the increasing of curing time which indicate that the viscoelasticity of the gel was enhanced relevantly due to the stronger cross-linking degree.

As the hydrogel tissue adhesives were used to promote the closure of damaged tissues, they should not only have reliable moisture bonding ability, but also be able to maintain the stability of the gel for a long time under the human body temperature. As shown in Figure 5C, the effect of temperature change (25–55 °C) on the rheological properties of CS-TBA-Fe hydrogels was investigated. The CS-TBA-Fe hydrogels with curing times of 0.5 h, 2 h and 12 h exhibited very stable rheological properties when the temperature was slowly increased from 25 °C to 55 °C. G’ was always larger than G’’, which indicated that the hydrogel had good stability in a warm and humid environment.

### 3.5. Self-Healing Ability of CS-TBA-Fe Hydrogels

**Figure 6:**
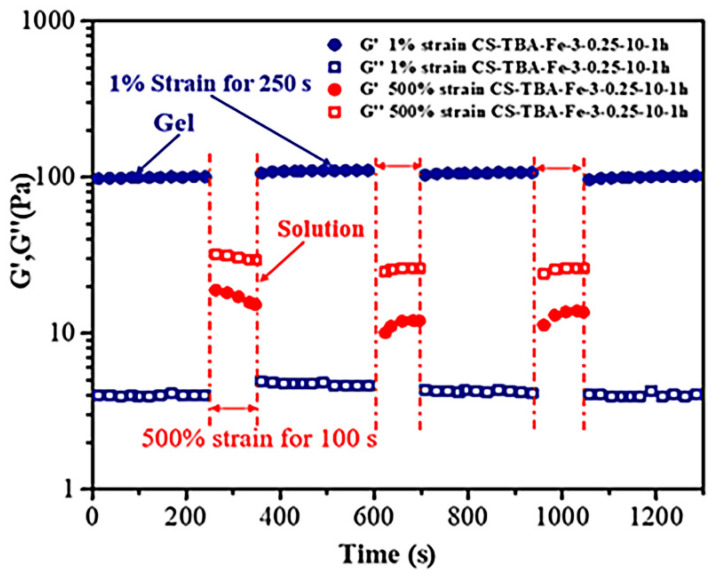
Self-healing ability of CS-TBA-Fe hydrogels under variable external strain (1% and 500%).

The alternate step strain sweep between 500% and 1% at the same frequency (1 Hz) was performed to assess the strain-induced damage and self-healing ability of the CS-TBA-Fe hydrogel (Figure 6). The time sweep test was first carried out at 1% strain to ensure that the sample was fully cross-linked. Then, a 500% strain was suddenly applied, and the G’ of the sample dropped from 105 Pa to 15 Pa and below G’’, indicating a complete disruption of the cross-linked state. Interestingly, once returned to a 1% strain, G’ immediately returned to the initial state. The disruption and recovery process of the CS-TBA-Fe hydrogel could be repeated alternately several times. The self-healing ability of CS-TBA-Fe hydrogels is stabler than CHI-C/DACNC hydrogels prepared by catechol-conjugated chitosan (CHI-C) and dialdehyde cellulose nanocrystal (DACNC) which can only recycle twice under the alternate step strain sweep [30]. The results indicated that the CS-TBA-Fe hydrogel had a fast and efficient self-healing ability.

### 3.6. Adhesion Strength of CS-TBA-Fe Hydrogels

**Figure 7:**
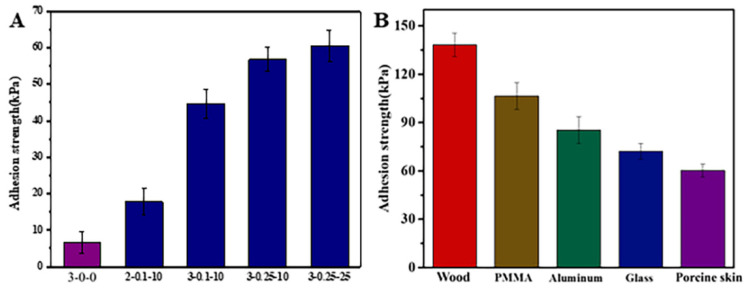
Adhesion strength of CS-TBA-Fe hydrogels with different structures on porcine skin (**A**) and comparison of the adhesion strengths in bonding various substrates (**B**).

The wet adhesiveness of the CS-TBA-Fe hydrogel was evaluated in vitro using a porcine skin so as to test its potential in tissue adhesion. As shown in Figure 7A, when the concentration of chitosan was increased from 2 wt% (2-0.1-10) to 3 wt% (3-0.1-10), the adhesive strength of CS-TBA-Fe hydrogels increased significantly from 17.9 kPa to 44.5 kPa. When the concentrations of TBA and Fe^3+^ increased to 0.25 M and 25 mM, the adhesive strength further increased to 60.3 kPa. These results indicate that the adhesive strength of the CS-TBA-Fe hydrogels was improved when increasing the concentration of amino groups, TBA and Fe^3+^ due to the higher cross-linking degree. Fibrin glue is a commercially used medical adhesive in common, and the tissue adhesion strength was about 5 kPa [31]. The CS-TBA-Fe-3-0.25-25 hydrogel exhibited ~12-fold enhanced wet tissue adhesion strength (60.3 kPa) over the commercial fibrin glue. Compared to another chitosan-based hydrogel CCOD-MgO adhesive [32] which was prepared by adding MgO to catechol-modified chitosan (CHI-C) and oxidized dextran (ODex), the CS-TBA-Fe adhesive presented nearly twice the adhesive strength.

Then, the adhesive strength of CS-TBA-Fe hydrogels on wood, PMMA, aluminum and glass were further quantified to reflect the surface suitability of the hydrogels and to broaden their potential application. As shown in Figure 7B, the adhesion strength values of CS-TBA-Fe-3-0.25-25 adhesives on various materials were arranged in descending order. Among the different materials, wood exhibited the highest adhesion strength (138.2 kPa). In addition, CS-TBA-Fe adhesives achieve adhesion strengths of 106.4 kPa, 85.3 kPa, and 72.1 kPa for PMMA, aluminum, and glass, respectively. The excellent surface adaptability allows the CS-TBA-Fe adhesive to be used not only as a tissue adhesive, but also for the purpose of adhere other surfaces.

### 3.7. Antibacterial Activity of CS-TBA-Fe Hydrogels

The amino groups in chitosan, pyrogallol moieties, and imine groups formed by Schiff-base reaction was supposed to give the hydrogel antibacterial capabilities [31]. The inhibitory effect of CS-TBA-Fe hydrogel on *E. coli* was first determined by optical density (OD) value. As shown in Table 2, the relative activity of *E. coli* was calculated from the OD values of the two samples at 600 nm. The relative activity of *E. coli* decreased significantly after co-cultured with CS-TBA-Fe hydrogel which indicates the good antibacterial ability of the hydrogel.

**Figure 8:**
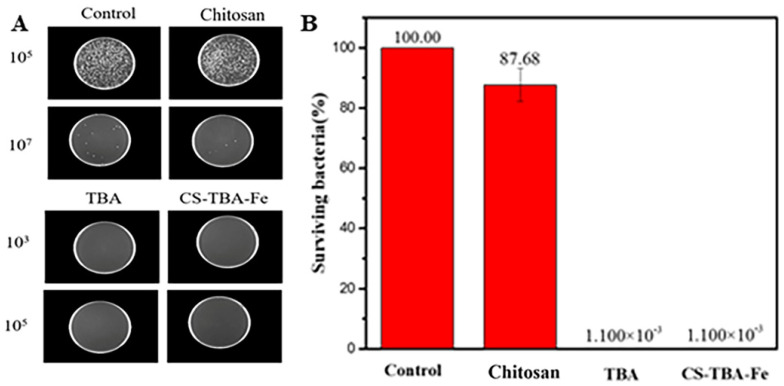
Digital images of surviving bacteria on chitosan, TBA, and CS-TBA-Fe (**A**) samples after being co-cultured with *E. coli* (A) and comparison of bactericidal activity of chitosan, TBA, and CS-TBA-Fe against *E. coli* (**B**).

The killing rates against *E. coli* of chitosan, TBA and CS-TBA-Fe hydrogel were further determined by spread plate coating method. The co-culture solution of each sample with *E. coli* was diluted with PBS solution firstly. After uniformly coating in lysogeny broth medium, the samples were finally incubated at 37 °C for 18 h. As shown in Figure 8, the TBA and CS-TBA-Fe hydrogel possessed about 99.99% killing rate against *E. coli*, which is much higher than that of chitosan (12.32%). These results indicated that the CS-TBA-Fe hydrogel exhibited an excellent antibacterial capacity mainly due to the existence of pyrogallol moieties in the hydrogel.

## 4. Conclusions

Inspired by tunicate adhesive protein, a novel biomimetic chitosan-based hydrogel was prepared by simply mixing chitosan-FeCl_3_ solution with TBA. The CS-TBA-Fe hydrogel showed milder preparation conditions, tunable gelation time and excellent tissue adhesion property. The hydrogel also exhibited strong adhesion to various substrates such as wood, PMMA, aluminum, glass and porcine skin. Additionally, the hydrogel possesses excellent self-healing ability and antibacterial property. Thus, all these capabilities make the multifunctional CS-TBA-Fe hydrogel a promising biomimetic material for various biomedical applications. Especially, we envision it will become a promising candidate as a tissue adhesive or wound dressing in an emergency situation.

## Figures and Tables

**Table 1:** Gelation time of CS-TBA hydrogels with different formula.

CS-TBA Formula	Gelation Time
CS-TBA-Fe-1-25-0	not gel
CS-TBA-Fe-2-15-0	10 h
CS-TBA-Fe-2-25-0	2 h 11 m
CS-TBA-Fe-3-25-0	21 m 31 s
CS-TBA-Fe-3-25-0 (0.2M HCl)	not gel

**Table 2:** Determination of the relative bacterial activity of *E. coil*.

Sample	OD_600_			Average	Bacterial Activity (%)
Control	0.0903	0.0789	0.0812	0.0835	10.6
CS-TBA-Fe	0.778	0.783	0.788	0.783	

## Data Availability

Not applicable.
